# No common understanding of profession terms utilized in health services research: An add-on qualitative study in the context of the QUALICOPC project in Austria

**DOI:** 10.1007/s00508-016-1146-y

**Published:** 2016-12-19

**Authors:** Kathryn Hoffmann, Silvia Wojczewski, Diederik Aarendonk, Manfred Maier, Thomas Ernst Dorner, Jan de Maeseneer

**Affiliations:** 1 Department of General Practice and Family Medicine, Center for Public Health, Medical University of Vienna, Kinderspitalgasse 15/I, 1090 Vienna, Austria; 2 European Forum for Primary Care, Utrecht, The Netherlands; 3 Institute for Social Medicine, Center for Public Health, Medical University of Vienna, Vienna, Austria; 4 Department of General Practice and Primary Health Care, Ghent University, Ghent, Belgium

**Keywords:** Health services research, Primary health care, Terminology, Health professions, Austria

## Abstract

**Background:**

Health services research, especially in primary care, is challenging because the systems differ widely between countries. This study aimed to explore the different understanding of the terminology used, particularly, regarding the professions nursing and medical secretaries.

**Methods:**

The study was an add-on study to the Quality and Costs in Primary Care (QUALICOPC) project in Austria and designed as qualitative research. The qualitative phase was conducted by using semi-structured telephone interviews with general practitioners (GP). and17 GPs participated in the study.

**Results:**

No uniform meaning of the terms commonly utilized for the abovementioned health professions could be found among Austrian GPs. For example, under the profession term practice assistants, nurses as well as literal medical secretaries with and without special education and related work competencies and responsibilities were subsumed.

**Conclusions:**

Our study results show that no uniform meaning of the terms commonly utilized for above described health profession could be found even within one country by GPs. These findings are highly relevant, especially, when trying to compare results with similar data from other countries or negotiating about workforce issues. Our findings implicate several action points for health services research and health policy. We propose the development of a harmonized terminology in Europe for the health profession based on standards of undergraduate and postgraduate education, competencies and continuous education commitments. This would not only benefit comparative health system research but also patient safety across Europe.

## Introduction

Primary care is becoming more and more popular worldwide because research over the last 40 years has provided evidence that strong primary care is associated with better health indicators, comparatively lower socioeconomic inequality and lower rates of unnecessary hospitalization [1–4]. Although there is ample evidence for the benefits of strong primary care systems, it is not completely clear which factors are of primary importance for these benefits; this makes further research in different primary care settings highly relevant [5–9]. Europe is an excellent laboratory to observe and assess different primary care systems which are located geographically close together [7, 10, 11]; however, the systems differ not only from a structural or organizational point of view but also in terms of their traditional terminology as well as their education and training systems for primary care [12–14]. Ideally, the same term should mean the same to the study participants as to persons who interpret the results of the study [12] but even in studies with a very profound and comprehensive questionnaire development process, such as the Quality and Costs in Primary Care (QUALICOPC) study [15] some terminology obstacles remain: while going through the initial findings of the Austrian part of the QUALICOPC project regarding primary care professionals it became obvious that the terminology used and translated did not seem to be the same for the participating GPs. Therefore, we performed an add-on study which aimed to analyze the scope of differences in terminology used by GPs for selected primary care professions, namely nurses and medical secretaries working in the primary care sector in Austria. Additionally, we analyzed the term “independently” in relation to the tasks and responsibilities of nurses and medical secretaries. Does it mean the person simply performs the task by alone (by order of someone who is then subsequently accountable for the outcome) or does it mean the individual performs the task autonomously and is, additionally, accountable for it? It is not the pure linguistic focus that the paper wants to add, it has to do with the complex also cultural intertwining of the way health systems and health services are shaped in Europe, and the words we used to indicate certain professionals in those services and systems.

## Methods

### Design

The study was an add-on study to the QUALICOPC project and was designed as a qualitative research [16, 17]. The design and recruitment of the overall QUALICOPC study and the Austrian part are described in depth elsewhere [15, 18–20]. Altogether, 184 GPs participated in the Austrian part of the study and the sample was roughly equally distributed in relation to sex, age, and office location compared to the national GP sample [21]. For this qualitative add-on survey all Austrian GPs that had already participated in the quantitative QUALICOPC study were invited via email to participate in a semi-structured telephone interview. The aim was to recruit a minimum of 15 GPs to reach data saturation; in the end, after participation of 17 GPs data saturation was reached.

### Interviews

The semi-structured telephone interviews were conducted by using an interview guide. The interviews were handled from a single office at the Medical University of Vienna, recorded, and finally transcribed verbatim by the second author. The interviews were between 15 and 35 min long and were conducted in German. The interview guide was designed by the first author with questions about the understanding of the translated primary care professions by the GPs. Additionally, the GPs where asked to describe the primary health care (PHC) professions they collaborated with, what kind of tasks and responsibilities these PHC professionals perform, and how the cooperation functions.

### Data analysis

A qualitative content analysis was performed with the software ATLAS.ti using an open inductive coding approach [22, 23]. ATLAS.ti is one of a new generation of qualitative data analysis software packages. This software packages can be used to analyze interviews, field notes, textual sources, and other types of qualitative data (http://atlasti.com/). The first and second author independently coded the interviews and compared and summarized their codes afterwards. We calculated the percentage of agreement for each code to assess intercoder reliability. A percentage agreement for the two coders (KH, SW) of more than 85% was achieved for all the codes. Categories and codes were inductively assigned based on the revision of the material as well as deductively based on the structure and themes of the interview guidelines. For the purpose of this publication the quotes were translated into the English language by the same two authors.

### Ethical considerations

The GPs had to give written informed consent before their participation. The QUALICOPC study and this analysis for Austria were approved by the Ethics Committee of the Medical University of Vienna (EC #808/2011).

## Results

### Understanding of the terminology used for primary care professions

In the telephone interviews, we asked the GPs which kind of health professions they worked with in their practice. Most of them used the term “practice assistants (*OrdinationsassistentIn*)” when answering this question. While scrutinizing what kind of professions they meant by “practice assistants” it became obvious that different kinds of professions with different kinds of education were meant; however, the majority meant medical secretaries and nurses.

- (P [participants]4:S [section]9) Two practice assistants work with me. […] One is a nurse and the other one has the training as a medical secretary.
- (P5:S8) I have two practice assistants, they are both nurses.
- (P17:S9) I only employ practice assistants who passed the special training course for medical secretaries of the Austrian chamber of physicians, that’s it.
- (P3:S11) I employ four practice assistants. Two of them have the training course as medical secretaries and the other two work as secretaries and cleaners.
- (P9:S17) My wife and a second practice assistant.

To clarify what level of underlying education the medical secretaries in the GPs offices had, we especially asked about it: In general, the training/education of the medical secretaries was very diverse: most of them did or still have to do the special training course for medical secretaries which is obligatory for medical secretaries only since January 2014 [24]. In this study their educational background reached from finished lower secondary school (after 9 years of education) or secondary school (after 12 years of education) only, to kindergarten teacher, social worker or nurse. Special for some respondents was that the GP employed persons as medical secretaries who had a training/education as a nurse.

Additionally, we asked the GPs what they understood in relation to the term *Krankenschwester/-pfleger* (nurse) which was asked in the QUALICOPC study. In general, we found that the meaning was not the same for all GPs. They associated different education levels and some respondents meant that the term was not up to date anymore, which is partly true as the correct Austrian term for nurse would be *diplomierte Gesundheits- und KrankenpflegerIn*; however, due to the length of the term the majority use *Krankenschwester/-pfleger* in the daily vernacular.

#### Krankenschwester/-pfleger

The majority understood the term as graduated nurse.

- (P3:S57) I would think of a graduated nurse. But to my knowledge the correct German term is “*Diplomierte Gesundheits- und KrankenpflegerIn*”.
- (P15:S73) *Krankenpfleger, Krankenschwester, Krankenpflegerin*, these are terms for graduated nurses.

However, some GPs associated this term with persons who care for patients without being educated as a nurse, such as carers for elderly persons or auxiliary nurses.

- (P5:S88) In this day and age one would use this word for persons that care for patients without being a graduated nurse as, for example, persons from the home help service or auxiliary nurses but not for graduated nurses.
- (P14:S64) Auxiliary nurses, they differ from graduated nurses, have a shorter education/training, more social education and are also legally not allowed to perform as many tasks as nurses.

### Understanding of the term “independently”

When we asked in the interviews what tasks the “practice assistants” were performing “independently” there were always a lot of answers. Asking more specifically if those tasks were delegated responsibilities or were performed autonomously, the GPs always answered that all task were delegated by her/him, except for administrative ones.

- (P17:S23) They write the electrocardiogram (ECG), perform lung function testing, do the physical therapy, […] prepare infusions and injections, assist with taking blood samples and sometimes do it on their own, […] under my supervision, but they do it independently […] Yes, that are all tasks that I delegate, also supervise, but which are performed independently by my staff.

Since we knew from the previous interview question that GPs meant medical secretaries and nurses with the term practice assistants, we specifically asked if the nurses had additional responsibilities when compared with the medical secretaries. The nurses were most of the time allowed to perform more tasks then the medical secretaries; however, sometimes they also performed the same tasks. What all had in common was that they performed the tasks not autonomously.

- (P2:S10) The nurse is also responsible for administrative work but compared to the medical secretaries she also performs blood sampling and a lot of wound care because we have a lot of patients with ulcus cruris. […] Of course, I take a look at each ulcus cruris before she does the wound care.
- (P9:S17) Primarily, she is doing administrative tasks and reception of patients but also renewals of continuous prescriptions and physical therapy.

We asked the GPs specifically about their employee’s involvement in vaccination, routine follow-up of patients with chronic diseases and wound care. Most GPs were doing the vaccinations, routine follow-up and wound care on their own as they said it is their obligation; the practice assistants assisted with weight and height measurements or lifestyle counselling and took blood samples as a delegated task. In some cases the nurses were allowed to do the vaccinations or wound management (P2:S34; P15:S20), but also delegated by the GPs which means in the end the GP is accountable.

- (P4:S35) No, I do the vaccinations on my own; as it is a private service I cannot let my assistants perform it.
- (P15:S20) Blood sampling, further, under my supervision performing injections, vaccinations, changing bandages, swabs, for example throat swabs, sterilization […] Obviously those are all tasks that I delegate.
- (P5:S10) I have two assistants, both nurses […] they have the training for wound care management and are responsible for wound care treatment but under my supervision.
- (P11:S8) They support the disease management program for type II diabetes […], coordinate for example the consultations with the ophthalmologists or perform blood samplings and ECGs.

## Discussion

To our knowledge this is the first study that investigated the understanding by GPs of the different meaning, underlying education, and professional responsibilities of different health professions. Surprisingly, not much research on these important terminological/semantic aspects for health services research has been done so far. The results show that no common understanding of the terms for the selected health professions could be found among Austrian GPs; furthermore, in relation to task performance for all of them the term “independently” meant responsible by order/delegation. It became obvious that some GPs meant different professions with different underlying pregraduate and postgraduate training when talking about *Krankenschwester/-pfleger* which ranged from auxiliary nurses to graduated nurses. One reason could have been the use of the vernacular word for this profession, such as was done in the QUALICOPC study instead of the correct professional term. In general, all nurses in Austria are graduated nurses. The minimum requirement to become a graduated nurse is a successfully completed low secondary level of education (9 years of school education) plus 2 further school years. The professional education comprises 4600 h of theoretical and practical courses within 3 years. The entire education and training takes place in the hospital sector; no special education for the ambulatory sector is available. Graduated nurses in Austria are not allowed to make diagnoses, order diagnostic tests or prescribe medications. With the exception of tasks, such as how to wash or bed a patient which they do autonomously they work under supervision and/or by order of physicians [25], however, it is planned that nurses should receive more autonomy in patient care within the coming years [26].

When it came to the description of the staff in their offices, the situation was even more diverse; the majority of staff members were subsumed by the GP under the term practice assistant *OrdinationsassistentIn*, although in reality nurses and medical secretaries, two different professions, were meant. The term *OrdinationsassistentIn* is the correct Austrian term for a medical secretary. To become a medical secretary in Austria it is obligatory since the year 2014 to successfully complete a training course. Before 2014 anybody could become a health secretary who was at least 18 years old and successfully completed 9 years of school education. This training course is offered by the Austrian Chamber of Physicians [27] or other institutions of further education and takes 1.5 years with overall 650 h of theoretical and practical courses in ambulatory care. The requirements are an age of 18 years and older and to have accomplished at least 9 years of school education; 4 years of elementary school and 5 years of lower secondary school. After this training medical secretaries are allowed to perform simple clinical tasks under supervision and by instruction of a physician or a nurse, such as taking blood samples, point of care testing, or helping with wound care.

All of them (nurses and medical secretaries) were not performing medical tasks autonomously but only under supervision of the GPs. The majority of the nurses did administrative tasks too and the medical tasks that were delegated by the GPs depended very much on what the GP was willing to or wanted to delegate. An example is vaccination; some GPs saw this as their own duty, some delegated this task to a nurse.

In general, for a GP the working process is substantially different depending on whether the nurse works autonomously or not. If a nurse is working by order only the GP still needs time to think about the patient’s complaints, to give the right order, and to check if everything was done correctly because the GP has the final legal responsibility. If a nurse is working autonomously, the GP can concentrate on other complex and difficult patient cases while the nurse is doing, e. g. all the chronic care visits independently at the same time. When considering the patients side there are studies showing that patient satisfaction and quality of care is the same or better if nurses take over certain tasks from physicians autonomously [28–30]. On the other hand, one recent U.S. study found that those organizational cultures that emphasized collegiality and quality but not autonomy were related to quality evaluation and improvement [31].

These results point in the direction that the terminology reflects the hierarchical employment situation. Both nurses and medical secretaries perform tasks under supervision only, which leads to the assumption that physicians might tend to consider and name both as helper/assistant *OrdinationsassistentInnen* despite different professional competencies and related earnings. Although, in general, in general practice nurses receive a higher salary than a medical secretary, it is possible to employ and pay a nurse as medical secretary. In Austria, both professions have to be employed by the GP who has to be self-employed and income is based on a mixed reimbursement model, with fee for services as the predominant financial system in primary care [32, 33]. This payment scheme in general practice could hinder the real shift of tasks from physicians to nurses. This becomes clear for example with home visits: Austrian GPs get paid for home visits only if they do it themselves. Besides the legal aspect it is, therefore, economically not feasible for a GP to employ a nurse who is doing the home visits; the GP would not get remunerated but would have to pay a nurse for that service [32, 33].

Bearing in mind this diversity in understanding on a national level, it becomes even more challenging when Austrian results are to be compared to data, for example, from the UK. In the UK practice nurses have a completely different undergraduate and postgraduate education as they have different tasks and autonomy in relation to patient care. Depending on the level of education, a practice nurse in the UK autonomously performs diagnostics and prescriptions of medications besides others patient consultations [34]. It could be speculated that GPs from countries where nurses perform some tasks on a daily base autonomously, understand the term “independently” differently.

The situation in relation to “medical secretaries” is similar. In Norway for example, medical secretaries need to have at least completed the secondary level of education (12 years of school education) and the education and training takes 3 years [35].

Our findings show that terminology matters when it comes to research in primary care, at both the national and international level. This observation informs several recommendations regarding comparative research in health services:

1. Interpretation of quantitative data about health professions should only be performed if the full context (pregraduate and postgraduate education, tasks and responsibilities performed autonomously or by order) of the professions in question as well as the understanding of the terms used in the related countries are known. For that, however, much more qualitative research would be needed. The interpretation of comparative international results without all the background knowledge could lead to profound misinterpretations: if researchers from different countries or political stakeholders talk about primary care professions, such as medical secretaries or nurses for example, there is a real danger that they assume that they are talking about the same profession but they are actually not. This can be a threat in relation to negotiation processes about international standards or responsibilities for different health professions resulting in different health care standards, workforce development ideas and differences in the resulting safety standards for patients worldwide.
2. A list of standard terms for health care professions should be introduced similar to the list drawn up by the European Pharmacopoeia Commission [36]. This list should contain not only the different terms but also the educational requirements of the professions, key aspects of professional education as well as tasks and responsibilities (delegated and autonomous). This list should then be obligatory for all comparative questionnaire surveys in relation to health care professions. But it is not only about the terms, the goal should be to harmonize the underlying education and training systems from school to university with respect to special needs in certain countries to really adjust health care standards to the best safety standards possible in health care.

A strength of the study is the qualitative approach which allowed a more in depth analysis of the ensuing findings. A limitation could be a possible selection bias of the participating GPs as their inclusion was voluntary; however, if taking into consideration that mainly highly motivated and reflective GPs participated in the study, it can be speculated that the answers would be even more diverse in other GP samples. In addition, it has to be considered that we interviewed GPs only. It would have been interesting to interview nurses and health secretaries too since the terminology about these profession groups were explored in this study.

## Conclusion

No homogeneous understanding for terms commonly used for health professions could be found among Austrian GPs. In contrast, it was clear for Austrian GPs that the term “independently” in respect to nurses and medical secretaries meant that these assistants would perform tasks by themselves but always on the order of the GP and not autonomously. These findings are highly relevant when trying to compare results with similar data from other countries or negotiating about workforce development issues.

Our findings implicate several action points for health services research and health policy. We propose the development of a harmonized terminology in Europe for health professions based on standards of undergraduate and postgraduate education, competencies and continuous education commitments. This would not only benefit comparative health system research but also patient safety across Europe. Moreover, the basis for negotiations would be the same.
